# Predictors for complications and the removal of osteosynthesis material after mandibular fractures: a retrospective analysis

**DOI:** 10.1186/s12903-026-08112-0

**Published:** 2026-03-23

**Authors:** Simon Bigus, Tim Lukas Elter, Margrit Welter, Jan Oliver Voss, Nils Lucca Kern, Heilwig Fischer, Steffen Koerdt, Kilian Kreutzer, Max Heiland, Carsten Rendenbach, Claudius Steffen

**Affiliations:** 1 Department of Oral and Maxillofacial Surgery, Charité – Universitätsmedizin Berlin, Corporate Member of Freie Universität Berlin and Humboldt-Universität zu Berlin, Augustenburger Platz 1, Berlin, 13353 Germany; 2 BIH Charité Digital Health Accelerator Program, Berlin Institute of Health at Charité – Universitätsmedizin Berlin, Charitéplatz 1, Berlin, 10117 Germany; 3 BIH Biomedical Innovation Academy, BIH Charité Clinician Scientist Program, Berlin Institute of Health at Charité – Universitätsmedizin Berlin, Charitéplatz 1, Berlin, 10117 Germany

**Keywords:** Mandibular fractures, Open fracture reduction, Internal fracture fixation, Osteosynthesis, Postoperative complications, Hardware removal

## Abstract

**Background:**

This study aimed to identify factors leading to postoperative complications after open reduction and internal fixation (ORIF) of mandibular fractures. In addition, predictors associated with plate removal after surgery were identified.

**Methods:**

This retrospective single-center cohort study included adult patients who underwent primary ORIF of mandibular fractures between 12/2017 and 12/2019. Demographic and clinical data were extracted from medical records. The primary outcome was postoperative surgical site– and hardware-related complications, defined as wound healing disorder, surgical site infection, fistula formation, or plate exposure. The secondary outcome was removal of osteosynthesis material. Multivariable logistic regression analysis was used to identify independent predictors for both outcomes.

**Results:**

A total of 377 patients were included. Postoperative surgical site– or hardware-related complications occurred in 17.0% of patients. Osteosynthesis material was removed in 112 patients (29.6%) during follow-up. The most frequent indication for removal was patient request without medical complaints (48.6%), followed by sensory disturbance (20.7%) and infection (18.9%). In multivariable analysis, falls as trauma mechanism were associated with a lower risk of postoperative complications (*p* < 0.05). Younger age (< 65 years; *p* < 0.01) and the occurrence of any postoperative complication (*p* < 0.01) were independently associated with subsequent osteosynthesis removal.

**Conclusions:**

Plate removal is more likely in cases with complications and in patients < 65 years. Further research is needed to investigate whether and to what extent a second operation for osteosyntheses removal is really necessary.

**Supplementary Information:**

The online version contains supplementary material available at 10.1186/s12903-026-08112-0.

## Background

Mandibular fractures are among the most frequent facial injuries encountered by facial trauma surgeons, with twice the occurrence rate of midfacial fractures and therefore an almost everyday occurrence in the field of oral and maxillofacial surgery [1–3]. Although injury patterns and mechanisms vary considerably within a given population, young males are most commonly affected [4]. Depending on the type and severity of the fracture, there are different treatment options [5, 6]. The management of most fractures requires surgical intervention, and postoperative complication rates have been documented to range from 6.6% to 21.2% [7–9].

A variety of factors have been previously identified as independent risk factors for postoperative complications. These include time from injury to treatment, tobacco use, and dental extraction [9]. Meanwhile, other factors, such as patterns of alcohol consumptions, use of osteosynthesis plates, fracture location, comorbidities, complex fractures, and type of antibiotic used, could only be identified as risk factors in some studies [7, 10]. Consequently, there is still uncertainty concerning independent predictors for postoperative complications and multivariate analyses are lacking.

The necessity of plate removal remains a controversial debate. While infections often necessitate plate removal and subsequent surgical interventions, individual patient considerations, such as discomfort without sensory disturbances, must also be taken into account [11–13]. Besides patients’ burden, negative side effects of these additional surgeries are expenses for the healthcare system and additional risk for peri- and intraoperative complications [14]. To this day, there remains a lack of sufficient data on predictors other than infection leading to the removal of osteosynthesis, particularly in adult patients [11–13]. Increased knowledge on indications for plate removals after open reduction and internal fixation (ORIF) of mandibular fractures may enhance surgeon’s awareness of particular complications or patient characteristics predominantly resulting in the removal of the osteosynthesis material at a later stage. While postoperative complications after mandibular fracture fixation have been widely reported, data distinguishing medically indicated from elective osteosynthesis removal and identifying independent predictors of removal beyond infection remain limited. The present study addresses this gap by analyzing predictors of both postoperative surgical site–related complications and subsequent osteosynthesis removal in a large single-center cohort. By differentiating between complication-driven and elective removal, our findings may support more targeted risk minimization during initial fracture treatment and inform clinical decision-making.

The primary aim of this study was to identify predictors of postoperative surgical site– and hardware related complications following ORIF of mandibular fractures. Secondary outcomes focused on determining independent risk factors associated with subsequent plate removal.

## Methods

### Study design

A retrospective, observational single-center study was conducted at the Department of Oral and Maxillofacial Surgery, Charité – Universitätsmedizin Berlin, including patients treated between April 2017 and December 2019. Follow-up data were available until May 2022. Ethical approval was obtained from the local ethics committee (Ethikkommission der Charité – Universitätsmedizin Berlin, EA2/189/21). The study was designed and reported in accordance with the STROBE guidelines for observational studies.

### Study population

All consecutive adult patients (≥ 18 years) who underwent primary surgical treatment with ORIF of mandibular fractures at our institution and complete available demographic and clinical data during the study period were eligible for inclusion. Patients treated with closed reduction, non-surgical management, external fixation, or resorbable fixation systems were excluded. Further exclusion criteria were age < 18 years, initial treatment at another hospital, or incomplete demographic or clinical data. Missing data were treated with pairwise exclusion.

**Fig. 1:**
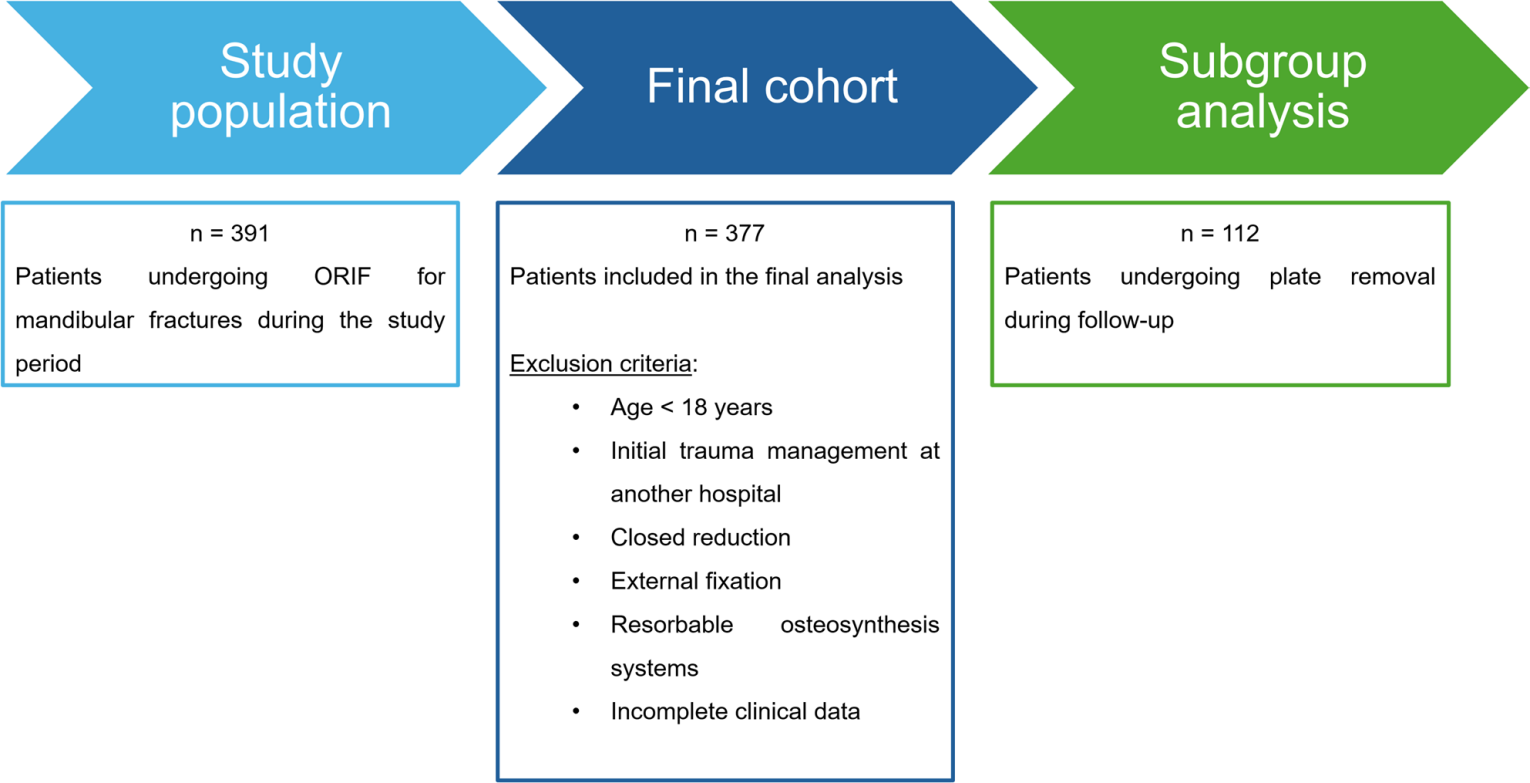
Flow of patients through the study including the final cohort with all eligible patients for statistical analysis and subgroup analysis of patients who underwent plate removal

Due to the institutional organization with a central emergency department and an independently operating medical care center using separate documentation systems, it was not possible to determine the absolute number of all patients with mandibular fractures during the study period. In addition, the ethical approval did not extend to cases managed exclusively by the independently operating medical care center. Patient inclusion, exclusion criteria, and allocation to the final study cohort and the subgroup undergoing osteosynthesis removal are summarized in Fig. 1.

### Perioperative management and follow-up

All eligible patients meeting the inclusion criteria were included. No a priori sample size calculation was performed due to the retrospective nature of the study. A post hoc power analysis (α = 0.05, power = 0.8) indicated a minimum required sample size of *N* = 33.37.

Perioperative management included preoperative clinical examination and three-dimensional (3D) imaging using cone-beam computed tomography (CBCT) or conventional computed tomography (CT). All patients received a single intraoperative dose of a broad-spectrum antibiotic (e.g., amoxicillin/clavulanic acid) in accordance with institutional standards. Postoperative antibiotic prolongation was performed in selected cases based on clinical risk factors such as soft tissue injury, tooth-bearing fracture sites, suspected infection, or relevant comorbidities.

Detailed information on postoperative antibiotic therapy, including mode of administration (none, intravenous, oral, or combined) and duration in days, was not available across all patients. Therefore these variables were not included as independent predictors in the main analysis but were instead explored in univariable analyses and multivariable sensitivity analyses. Postoperative thromboprophylaxis with low-molecular-weight heparin was administered to all hospitalized patients, adjusted to the individual risk profile.

Sensory disturbances and postoperative pain were initially managed conservatively according to institutional standards, including clinical observation, analgesic therapy, and—where appropriate—neuropathic pain medication. Plate removal was not routinely recommended for these symptoms alone but was considered on an individual basis after confirmed fracture healing.

Discharge was possible after 1–2 days with the first scheduled follow-up visit after 7–10 days. Follow-up duration and intervals varied depending on individual risk profiles and the occurrence of postoperative events; therefore, follow-up intervals were not standardized. Removal of osteosynthesis material was generally considered feasible after a minimum of six months postoperatively.

### Variables and data collection

Demographic and clinical variables extracted from medical records included sex, age at initial surgery, body mass index (BMI), trauma mechanism, fracture site, number of fracture sites, open versus closed fracture, type of osteosynthesis, duration of surgery, occlusion status, anticoagulation, diabetes mellitus, osteoporosis, nicotine abuse, alcohol consumption, postoperative surgical site–related complications, removal of osteosynthesis material, and mean follow-up time.

Age was analyzed both as a continuous variable and as a categorical variable (< 65 vs. ≥65 years) to explore potential threshold effects. The cutoff of 65 years was chosen based on clinical relevance in surgical risk assessment and exploratory data analysis [15]. Continuous variables (BMI, duration of surgery, follow-up time) were analyzed on their original scale, while categorical variables were analyzed as binary or nominal factors.

Initial osteosynthesis was performed using an intraoral approach whenever feasible. Extraoral access was reserved for specific indications such as condylar or complex fractures. As the surgical approach was strongly correlated with fracture pattern and complexity, it was not included as an independent variable in multivariate regression models to avoid multicollinearity and overadjustment.

Occlusion was assessed postoperatively based on clinical examination documented in the medical records and divided as partial dentate and fully dentate. Complete occlusion was defined as stable maxillomandibular contact without clinically relevant malocclusion requiring further intervention. Incomplete occlusion was defined as any documented deviation from normal occlusion.

Anticoagulation was defined as the use of anticoagulatory medication and/or platelet aggregation inhibitors in addition to routine postoperative thromboprophylaxis.

Soft tissue injury and antibiotic therapy were additionally analyzed as potential confounders. Soft tissue injury was documented as proximity to the fracture site (yes/no) and graded severity (four-level ordinal scale). Postoperative antibiotic therapy was recorded with respect to both mode of administration (none, intravenous, oral, or combined intravenous and oral) and duration in days. Due to incomplete documentation across the entire cohort and to avoid model overfitting, these variables were primarily explored in univariable analyses and subsequently included in sensitivity analyses of the multivariable regression models.

Data on the presence of teeth in the fracture line were not consistently documented and could therefore not be reliably analyzed.

### Primary outcomes

The primary outcome was the occurrence of postoperative surgical site– and hardware-related complications following ORIF. Complications were defined as any of the following events documented in the medical records from the index surgery until the last documented follow-up at our institution (end of follow-up May 2022): wound healing disorder, surgical site infection, fistula formation, or plate exposure.

Wound healing disorder was defined as postoperative wound dehiscence, delayed wound healing, or necrotic wound changes requiring additional clinical management, such as intensified wound care, prolonged local treatment, antibiotic therapy, or surgical revision. Minor postoperative findings not requiring clinical intervention were not classified as wound healing disorders. Surgical site infection was defined as a clinician-diagnosed infection requiring antibiotic therapy and/or surgical drainage or revision. Fistula was defined as a persistent mucosal or cutaneous tract with drainage or communication. Plate exposure was defined as visible exposure of osteosynthesis material through a mucosal or cutaneous defect.

Subjective postoperative symptoms such as sensory disturbances or postoperative pain were not included in the primary outcome, as they were inconsistently documented and may represent expected postoperative sequelae rather than complications requiring intervention. For the primary outcome, complications were analyzed as a binary variable (any complication: yes/no); patients with multiple events were counted once.

### Secondary outcomes

The secondary outcome was the removal of osteosynthesis material with the aim to identify independent risk factors associated with this event. Patients were categorized into a removal group and a non-removal group. For patients undergoing removal, additional variables were collected, including inpatient versus outpatient removal, length of hospital stay, extent of material removal, plate loosening, indication for removal (e.g., patient request, infection, sensory disturbance, pain, osteomyelitis, non-union), surgical approach, time interval from initial surgery to removal, and duration of the removal procedure.

### Statistical analysis

Statistical analysis was performed with R (Version 4.2.2, RStudio Team (2024), RStudio: Integrated Development for R, RStudio, PBC, Boston, MA). Data collection was performed using Microsoft Excel (Version 16.78.3., Microsoft Corporation, Redmond, USA).

Descriptive analyses were stratified according to clinical treatment characteristics and patient comorbidities (Additional file 1). Normal distribution of continuous variables was assessed prior to analysis. Student’s *t*-test (two groups) or analysis of variance (ANOVA; more than two groups) was applied for normally distributed continuous variables. Categorical variables were analyzed using the Chi-square test or Fisher’s exact test, as appropriate.

Multivariate logistic regression analysis was used to identify independent predictors of the primary and secondary outcomes. To avoid multicollinearity, subcategories of postoperative complications were not entered simultaneously into multivariate models. Variance inflation factors ranged between 1.0 and 2.0 for all included variables, indicating no relevant multicollinearity. Model significance was assessed using the Omnibus test of model coefficients, and explanatory power was evaluated using Nagelkerke’s R². Odds ratios (OR) with 95% confidence intervals (CI) are reported, but because of small subgroup sizes and low event numbers in certain categories, regression estimates may be affected by random variation and limited precision. A two-sided significance level of *p* < 0.05 was applied to all analyses.

## Results

### Study cohort

A total of 377 adult patients met the inclusion criteria and were included in the final analysis (75.9% male, 24.1% female). The flow of participants through the study, including reasons for exclusion and allocation to the subgroup undergoing osteosynthesis removal, is shown in Fig. 1.

### Patient and fracture characteristics

The mean age at initial surgery was 38.4 years (SD 17.4) and the mean BMI was 23.7 kg/m² (SD 4.4). Overall, 89.4% of patients were < 65 years at the time of initial surgery. Miniplates were used in 84.9% of cases, and assault was the most common trauma mechanism (47.0%). Nicotine abuse was documented in 41.4% of patients; diabetes mellitus in 4.5%, osteoporosis in 2.6%, and alcohol consumption in 2.4%. Detailed baseline characteristics are presented in Additional file 1.

Miniplates were the most frequently used osteosynthesis method in both age groups (< 65 and ≥ 65 years; 86.4% vs. 81.0%, *p* < 0.05). Among patients who subsequently required osteosynthesis removal, the second most common fixation method was a combination of miniplates and screw osteosynthesis (8.0%). In contrast, reconstruction plate osteosynthesis represented the second most frequently used method among patients who did not undergo plate removal (9.4%) (Additional file 1). Exclusive screw osteosynthesis was performed in three patients. In one case, an isolated symphyseal fracture was treated with lag screw fixation. In the remaining two cases, combined symphyseal and condylar head fractures were present; the symphysis was treated with lag screw osteosynthesis in both cases, while the condylar head fracture was surgically addressed in one patient and managed conservatively in the other due to the absence of fragment displacement.

### Primary outcome: postoperative surgical-site- and hardware-related complications

Overall, 17.0% of patients developed at least one postoperative surgical site– or hardware-related complication. The most frequent complication subtype was surgical site infection (8.9%), followed by wound healing disorder (6.7%), fistula formation (4.2%), and plate exposure (4.0%).

Patients who developed any postoperative complication were older than those without complications (44.1 ± 18.6 vs. 37.3 ± 17.0 years, *p* < 0.05). The exclusive use of miniplates was less frequent in patients with complications compared to those without complications (73.4% vs. 86.1%), whereas reconstruction plates were used more often in patients with complications (18.8% vs. 6.0%). Differences in trauma mechanisms between patients with and without complications were mainly attributable to less frequent causes. Assault (50.0% vs. 46.3%) and falls (14.1% vs. 28.4%) were the most common trauma mechanisms in both groups. Postoperative complications occurred more frequently in patients receiving anticoagulatory medication. Among these patients, 38.5% developed at least one complication. Specifically, 15.4% developed fistula formation, 11.5% surgical site infection, and 11.5% plate exposure. Anticoagulation (as defined in the Methods) was more frequent among patients with complications (15.6% vs. 5.1%, *p* < 0.01). Further stratified comparisons are shown in Additional file 1.

**Fig. 2:**
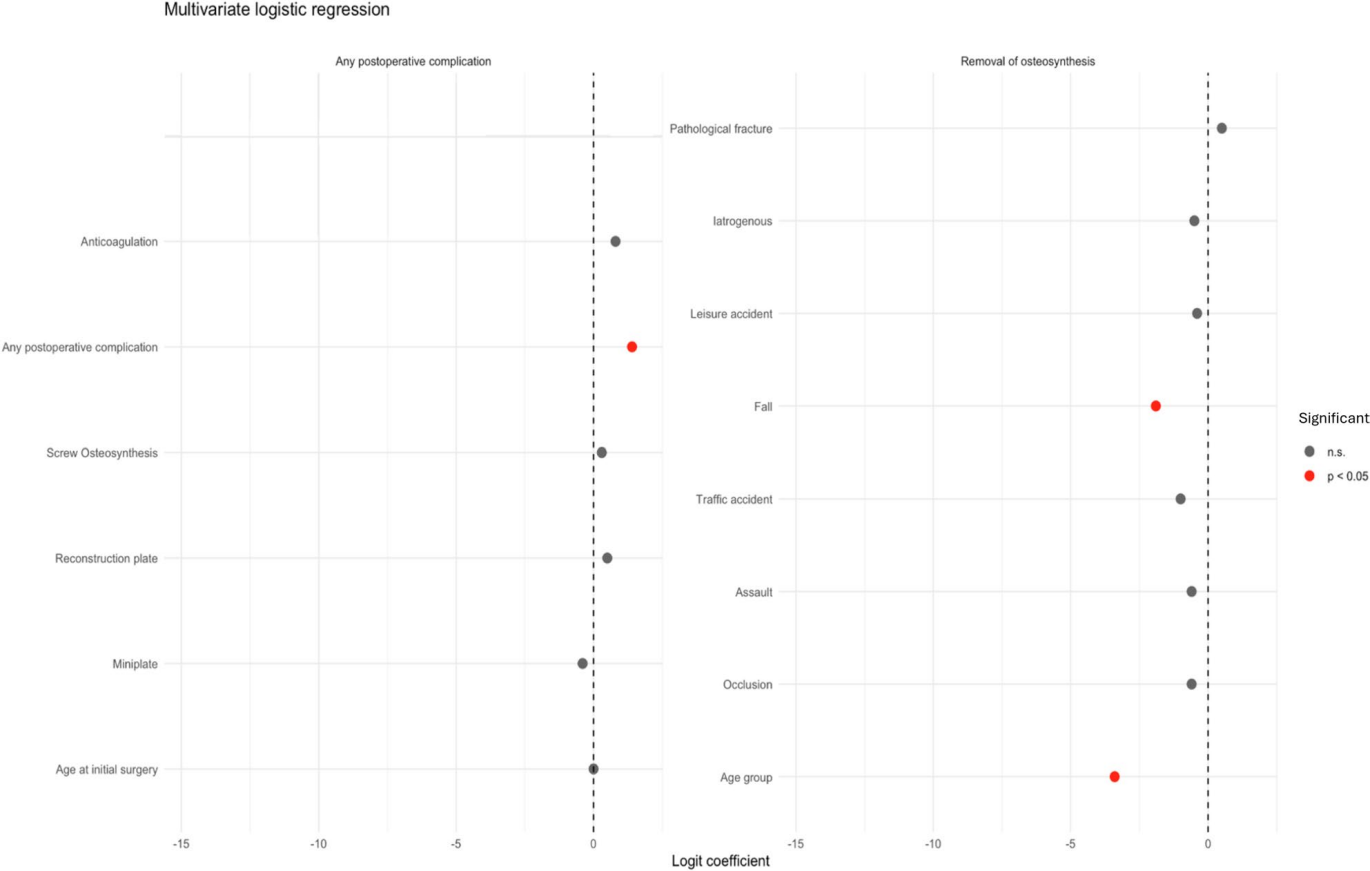
Depiction of logit coefficients for multivariate regression analysis for dependent variables any postoperative complication and removal of osteosynthesis. N.s = not significant. Significance is shown with a red dot

In multivariable analysis, falls as trauma mechanism were associated with a significantly lower risk of postoperative complications (OR 0.14, 95% CI 0.03–0.66; *p* = 0.02), whereas age and anticoagulation were not independently associated. The overall model was significant (Omnibus test *p* < 0.01) with Nagelkerke’s R² = 0.1 (Table 1; Fig. 2).

**Table 1:** Multivariate logistic regression analysis for primary outcome variable “any postoperative complications” and secondary outcome variable “Removal of Osteosynthesis”. Statistically significant values are highlighted. ORs with 95% CI are reported where model estimates were stable and clinically interpretable. Variables with small subgroup sizes or sparse data result in unstable estimates and wide confidence intervals. Estimates should be interpreted with caution due to limited statistical power and potential random effects

Multivariate logistic regression		Any postoperative complication			Removal of Osteosynthesis		
Variable		Logit coefficient	*P* - value	OR (95%CI	Logit coefficient	*P* - value	OR (95% CI)
	Age at initial surgery	0.0	0.25	1.01 (0.99–1.03)	.	.	.
	Age group	.	.	.	-3.4	< 0.01	0.06 (0.41–689.61)
	Occlusion	.	.	.	-0.6	0.23	0.60 (0.01–0.29)
Type of Osteosynthesis	Miniplate	-0.4	0.86	0.62 (0.04–89.94)	-6.1	0.35	0.07 (0.01–0.89)
	Reconstruction plate	0.5	0.85	1.31 (0.08–200.32)	-6.0	0.35	0.06 (0.01–0.89)
	Screw Osteosynthesis	0.3	0.85	1.41 (0.05–250.11)	-6.1	0.35	0.23 (0.01–0.89)
	Miniplate and Reconstruction plate	0.1.7	0.85	NA	-6.0	0.35	0.57 (0.01–32.33)
	Miniplate and Screw Osteosynthesis	-14.8	0.86	3.92 (0.13–768.32)	-6.1	0.35	0.31 (0.01–4.68)
	Reconstruction-plate and Screw Osteosynthesis	-14.7	0.85	1.04 (0.05–163.93)	-6.0	0.35	NA
Trauma mechanism	Assault	-0.6	0.45	0.49 (0.11–2.18)	.	.	.
	Traffic accident	-1.0	0.21	0.30 (0.06–1.49)	.	.	.
	Fall	-1.9	0.02	0.14 (0.03–0.66)	.	.	.
	Leisure accident	-0.4	0.72	0.67 (0.11–3.94)	.	.	.
	Iatrogenous	-0.5	0.60	0.65 (0.13–3.27)	.	.	.
	Pathological fracture	0.5	0.59	NA	.	.	.
	Any postoperative complication	.	.		1.4	< 0.01	3.83 (2.09–7.10)
	Anticoagulation	0.8	0.18	2.17 (0.74–6.24)	.	.	.
	Omnibus test	.	< 0.01	.	.	< 0.01	.
	Nagelkerkes R^2^	.	0.1	.	.	0.2	.

Soft tissue injury and postoperative antibiotic therapy were evaluated as potential confounders for postoperative surgical site– and hardware-related complications. Soft tissue injury near the fracture site was present in 57% of patients (*n* = 377). While the severity of soft tissue injury showed a significant association with postoperative complications in univariable analysis (*p* = 0.001), proximity of soft tissue injury to the fracture site demonstrated only a borderline association (*p* = 0.05). With respect to antibiotic therapy, neither the mode of postoperative antibiotic administration (*p* = 0.368) nor the duration of antibiotic treatment (*p* = 0.095) was significantly associated with the occurrence of postoperative complications. In sensitivity analyses including soft tissue injury proximity and antibiotic duration in the multivariable logistic regression model, neither variable remained independently associated with postoperative complications. Importantly, inclusion of these parameters did not materially alter the direction or magnitude of the main effects observed in the primary multivariable analysis.

### Secondary outcomes: removal of osteosynthesis material

During follow-up, 112 patients (29.6%) underwent removal of osteosynthesis material. The mean time from initial fixation to removal was 9.5 months (SD 6.2). Characteristics specific to the removal subgroup are summarized in Table 2.

**Table 2:** Descriptive analysis of characteristics which are specific for patients that got removal of osteosynthesis material in the further course

Variable	Description	Absolute frequency (relative frequency)
Osteosynthesis removal	N=	112
Hospitalization for Osteosynthesis removal	no	54 (48.6%)
	yes	57 (51.4%)
Duration of hospitalization (days)	mean (SD)^1^	1.4 (2.5)
Extent of Osteosynthesis removal	Total	87 (78.4%)
	Partial	13 (11.7%)
	Re-Osteosynthesis	11 (9.9%)
Plate loosening	no	89 (80.2%)
	yes	22 (19.8%)
Reason for Osteosynthesis removal	no complaints / patients` request	54 (48.6%)
	infection	21 (18.9%)
	sensory disturbance	23 (20.7%)
	pain	6 (5.4%)
	Osteomyelitis / non-union	7 (6.3%)
Surgical approach for Osteosynthesis removal	extraoral	10 (9.1%)
	intraoral	81 (73.6%)
	extraoral and intraoral	19 (17.2%)
Time from initial surgery to Osteosynthesis removal (months)	mean (SD)	9.5 (6.2)
Surgery time Osteosynthesis removal (min)	mean (SD)	73.9 (43.5)

The mean time from initial fixation to removal was 9.49 months (SD 6.18). Among patients undergoing implant removal, the mean follow-up time was 6.3 months (SD 9.5), with a range from 0 to 44.9 months. Removal was performed as an inpatient procedure in 51.0% of cases (mean length of stay 1.4 days, SD 2.5). Total removal was performed in 78.4%, partial removal in 11.7%, and re-osteosynthesis in 9.9%. An intraoral approach was used in 73.6% of cases.

The most frequently documented reason for osteosynthesis removal was patient request/no complaints (48.6%), followed by sensory disturbance (20.7%), infection (18.9%), pain (5.4%), and osteomyelitis/non-union (6.3%) (Table 2). Sensory disturbances and pain were documented as indications for removal in the respective subgroup; however, their incidence in the overall cohort could not be reliably assessed due to inconsistent documentation in patients without secondary surgery. The occurrence of any postoperative complication was independently associated with subsequent osteosynthesis removal (OR 3.83, 95% CI 2.09–7.10; *p* < 0.01).

### Comparisons between patients with and without osteosynthesis removal

Patients who underwent osteosynthesis removal were more frequently < 65 years compared to those without removal (99.0% vs. 85.3%, *p* < 0.01). Complete occlusion was documented more often in the removal group than in the non-removal group (94.0% vs. 84.2%, *p* < 0.05).

Postoperative surgical site– and hardware-related complications after the initial surgery were more frequent in the removal group than in the non-removal group (29.0% vs. 11.7%, *p* < 0.01). In particular, wound healing disorders (14.0% vs. 3.4%, *p* < 0.01), surgical site infections (15.0% vs. 6.0%, *p* < 0.05), fistula formation (11.0% vs. 1.5%, *p* < 0.01), and plate exposure (8.0% vs. 2.3%, *p* < 0.05) occurred more often among patients who later underwent removal (Additional file 1).

In multivariable logistic regression analysis for osteosynthesis removal, age group (< 65 vs. ≥65 years; *p* < 0.01) and the occurrence of any postoperative complication (*p* < 0.01) were identified as independent predictors. The overall model was significant (Omnibus test *p* < 0.01) with Nagelkerke’s R² = 0.2 (Table 1; Fig. 2).

## Discussion

This study investigated predictors of postoperative surgical site– and hardware-related complications following primary ORIF of mandibular fractures and, as a secondary outcome, independent predictors of subsequent removal of osteosynthesis material. While several risk factors for postoperative infection and wound complications have been described previously, the present analysis adds clinically relevant detail by differentiating predictors of surgical site–related complications from predictors of osteosynthesis removal and by highlighting the substantial proportion of removals performed for non-medical (patient-driven) reasons and the independent association with younger age. 

Overall, our cohort predominantly comprised younger male patients (89% under 65 years of age, 76% men), with assault as the most frequent trauma mechanism (46%), which is consistent with prior reports from comparable urban trauma populations [1, 4, 16]. This supports the representativeness of our study population with regard to typical mandibular fracture epidemiology.

### Postoperative surgical site– and hardware-related complications

Regarding the primary outcome variable (surgical site-related postoperative complications), only the trauma mechanism “fall” was identified as an independent risk factor associated with fewer postoperative complications. As this type of trauma predominantly occurs in older patients, one possible explanation is their higher compliance with postoperative care and rehabilitation protocols, which may reduce the risk of complications [17]. However, although age itself was not identified as an independent risk factor in the multivariate analysis, the univariate analysis demonstrated that patients who experienced postoperative complications after initial surgery were significantly older than those without complications (*p* < 0.02). This may indicate that older patients tend to adhere more consistently to postoperative recommendations; however, age-related factors such as comorbidities and reduced regenerative capacity may still contribute to an increased risk of complications [18]. In addition, anatomical changes commonly seen in elderly patients—such as altered dentition, a higher prevalence of atrophic mandibles, and the resulting shifts in biomechanical force distribution—may further impair bone healing [19]. Immunosenescence may also play a role: terminally differentiated effector memory CD8⁺ T cells (TEMRA cells), which are more prevalent in an “experienced” immune system and known for their highly pro-inflammatory profile, have been associated with impaired fracture healing [20].

Likewise, postoperative surgical site-related complications were more frequently noted in patients receiving anticoagulatory drugs. The influence of anticoagulants on bone healing is still being discussed in the literature. There is evidence that warfarin, heparin and aspirin delay fracture healing. Heparin and warfarin have an even greater detrimental impact than low-molecular-weight heparin [21]. Also unfractionated heparin leads to increased bone-resorption, through direct osteoclast stimulation [22]. According to the definition applied in this study, routine postoperative thromboprophylaxis with low-molecular-weight heparin, which was administered to all hospitalized patients, was not classified as anticoagulation. This decision was made because prophylactic heparin use did not allow for discrimination between patient groups, whereas long-term anticoagulation or platelet aggregation inhibitors were considered clinically relevant exposures. Nonetheless, the near-universal use of heparin among inpatients may have constituted an unaccounted confounding factor. Studies have shown a dose-independent reduction in osteoblast function after treatment with rivaroxaban in vitro [23]. On the other hand, it was indicated in vivo that these influences are negligible and do not result in a statistically significant negative effect on bone healing [24, 25].

Other factors influencing blood supply and bone healing, like nicotine abuse, are also often a matter of discussion and are linked to wound healing disorders and infections [26]. In our study, no significant difference could be demonstrated, despite nearly half of the patients being smokers (41%). Furthermore, an association between alcohol consumption and postsurgical complications in patients with mandibular fractures has been described. This correlation has been attributed not only to physiologic alterations but also to behavioral factors, including treatment adherence [9, 27, 28]. Nevertheless, neither alcohol consumption nor nicotine use showed a significant influence on postoperative complications in the present study. This is also the case for other previously discussed risk factors like, for example diabetes [29]. This discrepancy compared with previous reports may be explained by differences in cohort characteristics and variable operationalization. In particular, alcohol and nicotine consumption were recorded only as binary variables, precluding dose–response analyses, and postoperative complications were restricted to surgical site– and hardware-related events. Moreover, the relatively young study population and the low prevalence of severe comorbidities may have limited the ability to detect associations described in more heterogeneous cohorts.

The evidence behind the use of prophylactic antibiotics in mandibular fractures to prevent complications is weak [30]. 17.1% of patients in our cohort demonstrated any kind of postoperative complications after initial surgery although patients received antibiotics perioperatively. Perioperative antibiotic administration followed institutional standards. Detailed information on postoperative antibiotic therapy, including mode of administration and duration, was available for the majority of patients and was explored in univariable and multivariable sensitivity analyses. Neither the mode nor the duration of antibiotic therapy was independently associated with postoperative complications. Infections in general are the most common reason for the removal of osteosynthesis material (25%). As this is the main medical indication for the removal of osteosynthesis material, and therefore an influenceable variable, it has been investigated by other studies [28]. Ravikumar et al. highlighted infection as the main predictor leading to plate removal while wound dehiscence, occlusal disturbances and facial nerve paralysis were less common [31].

Moreover soft tissue injuries are frequently discussed as relevant risk factors for postoperative complications following mandibular fracture fixation. In the present cohort, soft tissue injury severity demonstrated a univariable association with postoperative complications; however, this effect did not persist after multivariable adjustment. Importantly, inclusion of these variables in sensitivity analyses did not change the main findings of the study, supporting the robustness of the primary results.

### Removal of osteosynthesis material

The results of this study showed that almost a third of the 377 patients included in this analysis underwent plate removal. 49% of patients opted for plate removal without the occurrence of postoperative complications, with no significant difference between the sexes (*p* = 0.74). There was a significant difference in terms of patient age. Patients under the age of 65 were more likely to have a second operation than patients older than 65 (*p* < 0.001), presenting patients’ age as an independent predictor for plate removals. This high rate of elective plate removal may in part be explained by institutional routines, as patients at our center are routinely informed about the option of plate removal following bone healing - even in the absence of symptoms. In addition, regional and national practices, including surgeon preference, patient counseling strategies, and health care system–specific factors such as reimbursement policies, may further influence the frequency of elective hardware removal [32–34]. Moreover, our data show that younger patients (< 65 years) were significantly more likely to request hardware removal.

Further explanation may be found in psychological motivation, aesthetic considerations or a more proactive approach to elective procedures among younger individuals. While our dataset did not allow direct quantification of psychological motivations, the literature highlights patient preference as a reason for elective plate removal. A meta-analysis corroborates that patient demand is among the most frequent indications for hardware removal, even in the absence of symptoms [35]. This aligns with the findings of Park et al., where the most frequent indication for removal was on patient demand (81.7%) [36]. Moreover, patients report subjective benefit from hardware removal, including relief from foreign-body sensations and psychological reassurance [37]. These psychological factors are recognized as valid indications for removal, and patients often express high willingness to undergo the procedure again if needed [38, 39]. In addition, aesthetic concerns may contribute to the decision for hardware removal, as miniplates can occasionally be palpable or may cause contour irregularities or associated with scarring, prompting some patients to request removal [40, 41]. Lastly, a generally more proactive approach to elective procedures among younger individuals is described [42]. However, these factors were not directly quantifiable in our dataset and should be regarded as potential explanations rather than evidence-based conclusions.

Sensory disturbances, including hypo- and hypersensitivity, were frequently reported among patients who underwent osteosynthesis removal and were documented as an indication for secondary surgery in 20.7% of these cases. These were not direct nerve damages, which already have been studied frequently in the literature, but rather the patient’s perception [43, 44]. As these symptoms were not systematically recorded in patients without secondary surgery, their incidence in the overall cohort could not be reliably assessed and they were not included in the primary outcome definition. Consequently, these findings should be interpreted as describing indications for removal rather than complication rates in the entire cohort.

### Osteosynthesis type and removal

The type of osteosynthesis could not be defined as an independent predictor for plate removal by the present study, nevertheless significant differences between the systems used and the performance of plate removals were found in the univariate analysis. Patients without removal of osteosynthesis were more frequently initially treated with reconstruction plates only. This is most likely due to the complexity of the fracture and the age of the patient. Reconstruction plates are used more frequently in complex fracture patterns, as well as in pathological fractures. The patient population of pencile-bone mandibular fractures consist mainly of older patients. In contrast, younger patients with less complex fracture patterns are preferably treated with miniplates and screws according to the AO guidelines (LIT). 

The high proportion of miniplate use in our cohort (84.9%) also reflects current practice in many European trauma centers. Similar patterns were reported by Aramanadka et al., highlighting that fixation preferences and institutional standards may influence removal rates and limit generalizability across surgical settings [11]. In this context, the use of miniplates rather than reconstruction plates and their potential stress-shielding effect of titanium osteosynthesis on bone remodeling should not be overlooked. Biomechanical and clinical data show that miniplates, due to their lower stiffness and reduced mass, transmit more physiological stress to the underlying bone, thereby reducing the risk of stress-shielding and subsequent bone resorption [45, 46]. Miniplates also demonstrate superior bone healing rates at 6 months postoperatively compared to reconstruction plates, with adequate fracture stability for new bone formation in non-comminuted fractures [46, 47]. Nevertheless, direct clinical evidence linking miniplates to stress-shielding–related adverse outcomes in mandibular fracture patients remains limited. Systematic reviews and retrospective series indicate that complications with miniplate fixation (e.g., infection, hardware failure, or removal) are not directly attributable to stress-shielding, and bone resorption or atrophy due to this mechanism is not well characterized [48, 49]. Thus, stress-shielding remains a theoretical concern with uncertain clinical significance in mandibular fracture fixation with titanium miniplates and remains an important area for future research.

Due to the young age of the majority of patients, careful and low-risk surgery is essential both from the patient’s point of view and the macroeconomic perspective.

### Clinical implications

From a clinical standpoint, these findings may help guide decision-making regarding osteosynthesis removal versus retention. First, the occurrence of postoperative surgical site– and hardware-related complications substantially increases the likelihood of later removal, emphasizing the importance of infection prevention, early detection, and consistent postoperative management.

Second, a large proportion of removals were elective and patient-driven, particularly among younger patients, highlighting the need for structured preoperative counseling regarding the expected course after ORIF, the potential benefits and risks of removal, and the uncertainty regarding routine versus selective removal strategies. While age itself did not emerge as an independent risk factor, age-related variables in the univariate analysis highlight the importance of comprehensive preoperative assessment and individualized management strategies. Infections were the primary indication for osteosynthesis removal, underscoring the clinical relevance of stringent infection control to reduce postoperative complications and avoid secondary surgery. The high proportion of elective plate removals, particularly among younger patients, further illustrates the strong influence of patient preference, emphasizing the need for thorough preoperative counseling.

Taken together, these findings can support clinical decision-making by identifying risk constellations in which plate removal is more likely. However its external validity is limited, and the true clinical benefit of routine versus selective removal remains uncertain. In addition, subjective postoperative symptoms such as sensory disturbance and pain were not systematically recorded in the entire cohort and were therefore not included in the primary outcome analysis. Prospective multicenter studies are needed to define clearer evidence-based recommendations.

### Limitations

This study has limitations inherent to its retrospective single-center design. Although several clinically relevant variables were available, including soft tissue injury characteristics and postoperative antibiotic therapy, incomplete documentation across the entire cohort limited their suitability for comprehensive multivariable adjustment. These variables were therefore explored in univariable analyses and sensitivity analyses, which did not materially alter the main findings. Other potentially relevant confounders could not be reliably extracted, including the presence of teeth in the fracture line, associated injuries and polytrauma status, frequency of postoperative visits, and patients’ distance to the treating facility, which may have introduced residual confounding. Postoperative compliance with behavioral recommendations and oral hygiene measures was not assessed. Moreover, the primary outcome was intentionally restricted to surgical site– and hardware-related complications. Other postoperative conditions such as malocclusion, paresthesia, postoperative pain, or non-union were not included, as these were not consistently documented in a standardized manner in the retrospective records or may represent expected postoperative sequelae rather than complications requiring intervention. Follow-up intervals were not standardized and varied according to clinical course, potentially limiting uniform detection of late events. Small subgroup sizes in several predictor categories may have resulted in imprecise regression estimates with wide confidence intervals, reflecting potential influence of random variation. In addition, the study focused on predictors of complications and osteosynthesis removal and did not assess outcomes of plate retention or long-term patient-reported outcomes. Alcohol and nicotine consumption were recorded only as binary variables, as quantitative data on amount and duration were not consistently available, precluding dose–response analyses. Prospective multicenter studies with standardized follow-up, structured documentation of relevant confounders, and systematic assessment of patient-reported outcomes are required to define evidence-based indications for osteosynthesis removal and to evaluate the safety and consequences of implant retention after mandibular fracture healing.

## Conclusions

In this retrospective single-center cohort, postoperative surgical site– and hardware-related complications were independently associated with subsequent removal of osteosynthesis material, and younger age emerged as an additional independent predictor of removal. Importantly, a substantial proportion of osteosynthesis removals were performed on patient request in the absence of documented surgical site–related complications, indicating that hardware removal is frequently not complication-driven. These findings highlight the relevance of patient-related factors in postoperative decision-making and underscore the importance of structured preoperative counseling and individualized follow-up strategies. While the results should be interpreted in light of residual confounding and non-standardized follow-up, the consistent findings across sensitivity analyses support the robustness of the main conclusions. Future prospective multicenter studies are warranted to establish evidence-based recommendations regarding selective versus routine osteosynthesis removal and to clarify the long-term clinical and patient-reported outcomes of implant retention following mandibular fracture fixation.

## Supplementary Information

Supplementary Material 1.

Supplementary Material 2.

## Data Availability

The datasets used and/or analyzed during the current study are available from the corresponding author on reasonable request.

## Abbreviations

ORIF: Open reduction and internal fixation
3D: Three-dimensional
CBCT: Cone beam computed tomography
STROBE guidelines: Strengthening the Reporting of Observational Studies in Epidemiology guidelines
ANOVA: Analysis of Variance
BMI: Body Mass Index (kg/m^2^)
SD: Standard Deviation
OR: Odds ratio
CI: Confidence intervals
TEMRA cells: Terminally differentiated effector memory CD8⁺ T cells
AO guidelines: All-in-one surgery reference guidelines

## References

[1] Afrooz PN, et al. The Epidemiology of Mandibular Fractures in the United States, Part 1: A Review of 13,142 Cases from the US National Trauma Data Bank. J Oral Maxillofac Surg. 2015;73(12):2361–6. 26006752 10.1016/j.joms.2015.04.032

[2] Haug RH, Prather J, Indresano AT. An epidemiologic survey of facial fractures and concomitant injuries. J Oral Maxillofac Surg. 1990;48(9):926–32. 2395044 10.1016/0278-2391(90)90004-l

[3] Ellis E, Moos KF, el-Attar A. Ten years of mandibular fractures: an analysis of 2,137 cases. Oral Surg Oral Med Oral Pathol. 1985;59(2):120–9. 3856795 10.1016/0030-4220(85)90002-7

[4] Erol B, Tanrikulu R, Görgün B. Maxillofacial fractures. Analysis of demographic distribution and treatment in 2901 patients (25-year experience). J Craniomaxillofac Surg. 2004;32(5):308–13. 15458673 10.1016/j.jcms.2004.04.006

[5] Pathak R, et al. Interventions for the Management of Mandibular Coronoid Process Fractures: A Systematic Review. J Maxillofac Oral Surg. 2023;22(2):433–41. 37122795 10.1007/s12663-022-01824-0 PMC10130277

[6] Nasser M, et al. Interventions for the management of mandibular fractures. Cochrane Database Syst Rev. 2013(7):Cd006087. 10.1002/14651858.CD006087.pub3 PMC11654902 23835608

[7] Furr AM, Schweinfurth JM, May WL. Factors associated with long-term complications after repair of mandibular fractures. Laryngoscope. 2006;116(3):427–30. 16540903 10.1097/01.MLG.0000194844.87268.ED

[8] Beckstrom TO, Dodson TB, Lang MS. Measuring Adherence to Antibiotic Use Guidelines in Managing Mandible Fractures. J Oral Maxillofac Surg. 2023;81(3):287–91. 36581312 10.1016/j.joms.2022.11.017

[9] Hsieh TY, et al. Risk Factors Associated With Complications After Treatment of Mandible Fractures. JAMA Facial Plast Surg. 2019;21(3):213–20. 30676610 10.1001/jamafacial.2018.1836 PMC6537826

[10] Malanchuk VO, Kopchak AV. Risk factors for development of infection in patients with mandibular fractures located in the tooth-bearing area. J Craniomaxillofac Surg. 2007;35(1):57–62. 17298884 10.1016/j.jcms.2006.07.865

[11] Aramanadka C, et al. Hardware Removal in Maxillofacial Trauma: A Retrospective Study. ScientificWorldJournal. 2021;2021:p9947350. 10.1155/2021/9947350 PMC8245212 34257626

[12] Islamoglu K, et al. Complications and removal rates of miniplates and screws used for maxillofacial fractures. Ann Plast Surg. 2002;48(3):265–8. 11862030 10.1097/00000637-200203000-00006

[13] Rallis G, et al. Reasons for miniplate removal following maxillofacial trauma: a 4-year study. J Craniomaxillofac Surg. 2006;34(7):435–9. 16963270 10.1016/j.jcms.2006.07.001

[14] Pontell ME, et al. Resorbable Versus Titanium Rigid Fixation for Pediatric Mandibular Fractures: A Systematic Review, Institutional Experience and Comparative Analysis. Craniomaxillofac Trauma Reconstr. 2022;15(3):189–200. 36081676 10.1177/19433875211022573 PMC9446277

[15] Al-Refaie WB, et al. Comparative Age-Based Prospective Multi-Institutional Observations of 12,367 Patients Enrolled to the American College of Surgeons Oncology Group (ACOSOG) Z901101 Trials (Alliance). Ann Surg Oncol. 2019;26(13):4213–21. 31605327 10.1245/s10434-019-07851-5 PMC6868343

[16] Goth S, Sawatari Y, Peleg M. Management of pediatric mandible fractures. J Craniofac Surg. 2012;23(1):47–56. 22337373 10.1097/SCS.0b013e318240c8ab

[17] Coulter JS, et al. Falls in Older Adults: Approach and Prevention. Am Fam Physician. 2024;109(5):447–56. 38804759

[18] Wolf S, et al. Surgical treatment, complications, reoperations, and healthcare costs among patients with clavicle fracture in England. BMC Musculoskelet Disord. 2022;23(1):135. 35139854 10.1186/s12891-022-05075-5 PMC8830003

[19] Zavlin D, et al. Multi-institutional Analysis of Surgical Management and Outcomes of Mandibular Fracture Repair in Adults. Craniomaxillofac Trauma Reconstr. 2018;11(1):41–8. 29387303 10.1055/s-0037-1603460 PMC5790542

[20] Jin R, et al. Impact of Age on Surgical Outcomes Following Mandible Fracture Repair. Laryngoscope. 2023;133(2):287–93. 35638520 10.1002/lary.30208

[21] Butler AJ, Eismont FJ. Effects of Anticoagulant Medication on Bone-Healing. JBJS Rev. 2021;9(5):e2000194. 10.2106/JBJS.RVW.20.00194 33999912

[22] Lindner T, et al. The effect of anticoagulant pharmacotherapy on fracture healing. Expert Opin Pharmacother. 2008;9(7):1169–87. 18422474 10.1517/14656566.9.7.1169

[23] Solayar GN, Walsh PM, Mulhall KJ. The effect of a new direct Factor Xa inhibitor on human osteoblasts: an in-vitro study comparing the effect of rivaroxaban with enoxaparin. BMC Musculoskelet Disord. 2011;12:247. 22035050 10.1186/1471-2474-12-247 PMC3215189

[24] Prodinger PM, et al. Does Anticoagulant Medication Alter Fracture-Healing? A Morphological and Biomechanical Evaluation of the Possible Effects of Rivaroxaban and Enoxaparin Using a Rat Closed Fracture Model. PLoS ONE. 2016;11(7):e0159669. 27455072 10.1371/journal.pone.0159669 PMC4959754

[25] Demirtas A, et al. Investigation of the effects of Enoxaparin, Fondaparinux, and Rivaroxaban used in thromboembolism prophylaxis on fracture healing in rats. Eur Rev Med Pharmacol Sci. 2013;17(14):1850–6. 23877846

[26] Ahmed A, et al. Potentially modifiable patient factors in mandible fracture complications: a systematic review and meta-analysis. Br J Oral Maxillofac Surg. 2022;60(3):266–70. 35183372 10.1016/j.bjoms.2021.07.005

[27] Serena-Gómez E, Passeri LA. Complications of mandible fractures related to substance abuse. J Oral Maxillofac Surg. 2008;66(10):2028–34. 18848098 10.1016/j.joms.2008.06.022

[28] Chen CL, et al. Complications and Reoperations in Mandibular Angle Fractures. JAMA Facial Plast Surg. 2018;20(3):238–43. 29302682 10.1001/jamafacial.2017.2227 PMC5876800

[29] Raikundalia M, et al. Facial fracture repair and diabetes mellitus: An examination of postoperative complications. Laryngoscope. 2017;127(4):809–14. 27658923 10.1002/lary.26270

[30] Dawoud BES, et al. Use of antibiotics in traumatic mandibular fractures: a systematic review and meta-analysis. Br J Oral Maxillofac Surg. 2021;59(10):1140–7. 34711441 10.1016/j.bjoms.2021.01.018

[31] Ravikumar C, Bhoj M. Evaluation of postoperative complications of open reduction and internal fixation in the management of mandibular fractures: A retrospective study. Indian J Dent Res. 2019;30(1):94–6. 30900664 10.4103/ijdr.IJDR_116_17

[32] Becker P, et al. Facial trauma management: A nationwide data collection on practice patterns and patient care in oral and maxillofacial surgery in Germany. J Craniomaxillofac Surg. 2025;53(7):999–1008. 40175198 10.1016/j.jcms.2025.03.013

[33] Bär AK, et al. Current orthognathic surgery practices: A comprehensive survey from planning to discharge in Oral and Maxillofacial Surgery. J Craniomaxillofac Surg. 2025;53(7):927–37. 40113459 10.1016/j.jcms.2025.03.004

[34] Shah JK, et al. Risk Factors for Hardware Removal Following Bimaxillary Surgery: A National Database Analysis. J Craniofac Surg. 2024;35(2):572–6. 38231209 10.1097/SCS.0000000000009929

[35] Jaber M, et al. Reasons for Removal of Miniplates Used in Fixation of Maxillofacial Bone Fractures: Systematic Review and Meta-Analysis. Appl Sci. 2023;13(21):11899.

[36] Park HC, et al. Mini-plate removal in maxillofacial trauma patients during a five-year retrospective study. J Korean Assoc Oral Maxillofac Surg. 2016;42(4):182–6. 27595084 10.5125/jkaoms.2016.42.4.182 PMC5009191

[37] Thorén H, et al. Symptomatic plate removal after treatment of facial fractures. J Craniomaxillofac Surg. 2010;38(7):505–10. 20199870 10.1016/j.jcms.2010.01.005

[38] Termer A, et al. Subjective outcomes and complication rates following elective implant removal: a prospective study on patient and surgeon perspectives. Arch Orthop Trauma Surg. 2025;145(1):334. 40468022 10.1007/s00402-025-05949-y

[39] Masoni V, et al. Implant removal: benefits and drawbacks - Results of a survey with five hundred participants from the Italian Society of Orthopedic Surgery and Traumatology (SIOT) and comparison with other international trends. Int Orthop. 2025;49(8):1775–87. 40415005 10.1007/s00264-025-06564-7 PMC12283862

[40] Jaber M, et al. Risk Factors Contributing to Symptomatic Miniplate Removal following Orthognathic Surgery: Systematic Review and Meta-Analysis. J Clin Med. 2024;13(11):3335. 38893045 10.3390/jcm13113335 PMC11172665

[41] Schmidt BL, et al. The removal of plates and screws after Le Fort I osteotomy. J Oral Maxillofac Surg. 1998;56(2):184–8. 9461142 10.1016/s0278-2391(98)90865-5

[42] Grillo R, et al. Should orthognathic surgery be performed in growing patients? A scoping review. J Craniomaxillofac Surg. 2023;51(1):60–6. 36658055 10.1016/j.jcms.2023.01.004

[43] Cillo JE Jr., et al. Neurosensory Recovery Following Mental Nerve Skeletonization in Intraoral Open Reduction and Internal Fixation of Mandible Fractures. J Oral Maxillofac Surg. 2021;79(1):183–91. 32961124 10.1016/j.joms.2020.08.027

[44] Tay AB, et al. Inferior Alveolar Nerve Injury in Trauma-Induced Mandible Fractures. J Oral Maxillofac Surg. 2015;73(7):1328–40. 25914133 10.1016/j.joms.2015.02.003

[45] Wongwaithongdee U, Inglam S, Chantarapanich N. Biomechanical evaluation of two internal fixation systems for the treatment of mandibular symphyseal fracture. Proc Inst Mech Eng H. 2023;237(5):597–606. 37070457 10.1177/09544119231168506

[46] Bohner L, et al. Treatment of Mandible Fractures Using a Miniplate System: A Retrospective Analysis. J Clin Med. 2020;9(9). 10.3390/jcm9092922 PMC7565660 32927782

[47] Steffen C, et al. Patient-specific miniplates versus patient-specific reconstruction plate: A biomechanical comparison with 3D-printed plates in mandibular reconstruction. J Mech Behav Biomed Mater. 2023;140:105742. 36857975 10.1016/j.jmbbm.2023.105742

[48] Capucha T, et al. Is Open Reduction Internal Fixation Using Titanium Plates in the Mandible as Successful as We Think? J Craniofac Surg. 2022;33(4):1032–6. 34608010 10.1097/SCS.0000000000008258

[49] Sejati BP, et al. Complications following miniplate insertion in maxillofacial fractures: a systematic review. F1000Res. 2024;13:p1507. 10.12688/f1000research.159017.3 PMC11869201 40028448

