# Supplementary material for: Predictors for complications and the removal of osteosynthesis material after mandibular fractures: a retrospective analysis
Source: BMC Oral Health. 2026 Mar 23;26:620. doi: 10.1186/s12903-026-08112-0 (PMC13063742; doi:10.1186/s12903-026-08112-0)
Supplement: Supplementary file 1 — Supplementary Material 1. [file 12903_2026_8112_MOESM1_ESM.docx]

**Supplementary materials**

*Title Table 2 – Included patients comorbidities and complications*

| **Variable** | **Category** | **Total (n=377)** | **No postop complication (n=313)** | **Postop complication (n=64)** | **P-value** | **No plate removal (n=263)** | **Plate removal (n=114)** | **P - value** |
| --- | --- | --- | --- | --- | --- | --- | --- | --- |
| **Postop complication** | No | 313 (83.0%) | 313 (100.0%) | – | – | 234 (88.3%) | 79 (71.0%) | **<0.01** |
|  | Yes | 64 (17.0%) | – | 64 (100.0%) |  | 31 (11.7%) | 33 (29.0%) |  |
| **Wound healing disorder** | No | 352 (93.3%) | 313 (100.0%) | 39 (61.0%) |  | 256 (96.6%) | 96 (86.0%) | **<0.01** |
|  | Yes | 25 (6.7%) | – | 25 (39.0%) |  | 9 (3.4%) | 16 (14.0%) |  |
| **Infection** | No | 344 (91.1%) | 313 (100.0%) | 31 (48.4%) |  | 249 (94.0%) | 95 (85.0%) | **<0.01** |
|  | Yes | 33 (8.9%) | – | 33 (51.6%) |  | 16 (6.0%) | 17 (15.0%) |  |
| **Fistula** | No | 361 (95.8%) | 334 (100.0%) | 48 (75.0%) |  | 261 (98.5%) | 100 (89.0%) | **<0.01** |
|  | Yes | 16 (4.2%) | – | 16 (25.0%) |  | 4 (1.5%) | 12 (11.0%) |  |
| **Plate exposure** | No | 362 (96.0%) | 313 (100.0%) | 49 (76.6%) |  | 259 (97.7%) | 103 (92.0%) | **0.02** |
|  | Yes | 15 (4.0%) | – | 15 (23.4%) |  | 6 (2.3%) | 9 (8.0%) |  |
| **Anticoagulation** | No | 349 (93.1%) | 295 (94.9%) | 54 (84.4%) | **<0.01** | 241 (91.6%) | 108 (96.0%) | 0.07 |
|  | Yes | 26 (6.9%) | 16 (5.1%) | 10 (15.6%) |  | 22 (8.4%) | 4 (4.0%) |  |
| **Diabetes** | No | 359 (95.5%) | 298 (95.5%) | 61 (95.3%) | 1.00 | 251 (95.1%) | 108 (96.0%) | 0.61 |
|  | Yes | 17 (4.5%) | 14 (4.5%) | 3 (4.7%) |  | 13 (4.9%) | 4 (4.0%) |  |
| **Osteoporosis** | No | 366 (97.4%) | 306 (98.1%) | 60 (93.8%) | 0.06 | 255 (96.6%) | 111 (99.0%) | 0.29 |
|  | Yes | 10 (2.6%) | 6 (1.9%) | 4 (6.2%) |  | 9 (3.4%) | 1 (1.0%) |  |
| **Smoking** | No | 194 (58.6%) | 162 (60.0%) | 32 (53.3%) | 0.41 | 134 (58.5%) | 60 (59.0%) | 1.00 |
|  | Yes | 137 (41.4%) | 109 (40.0%) | 28 (46.7%) |  | 95 (41.5%) | 42 (41.0%) |  |
| **Alcohol** | No | 368 (97.6%) | 305 (97.4%) | 61 (98.4%) | 0.99 | 257 (97.0%) | 111 (99.0%) |  |
|  | Yes | 9 (2.4%) | 8 (2.6%) | 1 (1.6%) |  | 8 (3.0%) | 1 (1.0%) |  |

*Table 2: Descriptive analysis of patients’ comorbidities and complications, stratified by presence or absence of plate removal and postoperative complications. Categorical variables were analyzed with Chi-square or Fisher’s exact test as appropriate. Significant p-values (p < 0.05) are highlighted.*
