# Supplementary material for: Predictors for complications and the removal of osteosynthesis material after mandibular fractures: a retrospective analysis
Source: BMC Oral Health. 2026 Mar 23;26:620. doi: 10.1186/s12903-026-08112-0 (PMC13063742; doi:10.1186/s12903-026-08112-0)
Supplement: Supplementary file 2 — Supplementary Material 2. [file 12903_2026_8112_MOESM2_ESM.docx]

**Additional File 1**. Patient characteristics stratified by postoperative complications and osteosynthesis removal

| **Variable** | **Category/Description** | **Total (n=377)** | **No postop complication (n=313)** | **Postop complication (n=64)** | **P-value** | **No plate removal (n=263)** | **Plate removal (n=114)** | **P-value** |
| --- | --- | --- | --- | --- | --- | --- | --- | --- |
| **Sex** | male | 286 (75.9%) | 235 (75.1%) | 51 (80.0%) | 0.45 | 200 (75.5%) | 86 (77.0%) | 0.85 |
|  | female | 91 (24.1%) | 78 (24.9%) | 13 (20.0%) | 0.45 | 65 (24.5%) | 26 (23.0%) | 0.85 |
| **BMI^1^** | mean (SD)^2^ | 23.7 (4.4) | 23.7 (4.6) | 23.5 (3.8) | 0.36 | 23.6 (4.9) | 23.7 (3.4) | 0.33 |
| **Age at initial surgery (years)** | mean (SD) | 38.4 (17.4) | 37.3 (17.0) | 44.1 (18.6) | 0.02 | 40.1 (18.7) | 34.3 (12.9) | 0.63 |
| **Age group** | < 65 | 337 (89.4%) | 281 (89.8%) | 56 (87.5%) | 0.81 | 226 (85.3%) | 111 (99.0%) | <0.01 |
|  | > 65 | 40 (10.6%) | 32 (10.2%) | 8 (12.5%) | 0.81 | 39 (14.7%) | 1 (1.0%) | <0.01 |
| **Initial surgery time (min)** | mean (SD) | 103.2 (74.6) | 102.6 (77.9) | 112.4 (76.0) | 0.87 | 102.1 (74.3) | 105.9 (75.6) | 0.26 |
| **Removal of Osteosynthesis** | no | 265 (70.3%) | 234 (74.8%) | 31 (48.4%) | . | 265 (71.0%) | . | . |
|  | yes | 112 (29.7%) | 79 (25.2%) | 33 (51.6%) | . | . | 112 (29.0%) | . |
| **Follow-Up-Time (days)** | Mean (SD) | 188.6 (258.1) | 173.4 (256.7) | 263.1 (256.0) | . | 104.3 (208.6) | 388.1 (256.2) | . |
| **Occlusion** | fully dentate | 328 (87.0%) | 275 (87.9%) | 53 (82.8%) | 0.25 | 223 (84.2%) | 105 (94.0%) | 0.01 |
|  | partial dentate | 49 (13.0%) | 38 (12.1%) | 11 (17.2%) | 0.25 | 42 (15.8%) | 7 (6.0%) | 0.01 |
| **Type of Osteosynthesis** | Miniplate | 320 (84.9%) | 273 (86.1%) | 47 (73.4%) | <0.01 | 229 (86.4%) | 91 (81.0%) | 0.04 |
|  | Reconstruction-plate | 31 (8.2%) | 19 (6.0%) | 12 (18.8%) | <0.01 | 25 (9.4%) | 6 (5.0%) | 0.04 |
|  | Screw-Osteosynthesis | 4 (1.1%) | 3 (1.0%) | 1 (1.5%) | <0.01 | 2 (0.8%) | 2 (2.0%) | 0.04 |
|  | Mini-plate and Reconstruction plate | 3 (0.8%) | 1 (0.3%) | 2 (3.1%) | <0.01 | 1 (0.4%) | 2 (2.0%) | 0.04 |
|  | Miniplate and screw-Osteosynthesis | 17 (4.5%) | 15 (4.7%) | 2 (3.1%) | <0.01 | 8 (3.0%) | 9 (8.0%) | 0.04 |
|  | Reconstruction-plate and Screw-Osteosynthesis | 2 (0.5%) | 2 (0.6%) | 0 (0.0%) | <0.01 | . | 2 (2.0%) | 0.04 |
| **Fracture site** | Condylar head and neck fracture | 4 (1.1%) | 4 (1.2%) | 0 (0.0%) | 0.06 | 4 (1.5%) | 0 (0.0%) | 0.21 |
|  | Corpus | 135 (35.8%) | 105 (33.5%) | 30 (46.9%) | 0.06 | 91 (34.3%) | 44 (39.3%) | 0.21 |
|  | Angle | 68 (18.0%) | 54 (17.3%) | 14 (21.9%) | 0.06 | 44 (16.6%) | 24 (21.4%) | 0.21 |
|  | Combination | 170 (44.8%) | 150 (p.7%) | 20 (31.2%) | 0.06 | 126 (47.5%) | 44 (38.4%) | 0.21 |
| **Trauma mechanism** | Assault | 177 (47.0%) | 145 (46.3%) | 32 (50.0%) | <0.01 | 128 (48.3%) | 49 (44.0%) | 0.05 |
|  | Traffic accident | 56 (14.9%) | 49 (15.7%) | 7 (10.9%) | <0.01 | 34 (12.8%) | 22 (20.0%) | 0.05 |
|  | Fall | 98 (26.0%) | 89 (28.4%) | 9 (14.1%) | <0.01 | 77 (29.1%) | 21 (19.0%) | 0.05 |
|  | Leisure accident | 19 (5.0%) | 15 (4.8%) | 4 (6.3%) | <0.01 | 10 (3.8%) | 9 (8.0%) | 0.05 |
|  | Iatrogenous | 17 (4.5%) | 10 (3.2%) | 7 (11.0%) | <0.01 | 10 (3.8%) | 7 (6.0%) | 0.05 |
|  | Pathological fracture | 10 (2.7%) | 5 (1.6%) | 5 (7.8%) | <0.01 | 6 (2.3%) | 4 (4.0%) | 0.05 |
| **Number of fractures** | Simple | 136 (36.1%) | 106 (33.9%) | 30 (46.9%) | 0.17 . | 94 (35.5%) | 42 (38.0%) | 0.67 |
|  | Double | 171 (45.3%) | 145 (46.3%) | 26 (40.6%) | 0.17 . | 124 (46.8%) | 47 (42.0%) | 0.67 |
|  | Triple | 58 (15.4%) | 52 (16.6%) | 6 (9.4%) | 0.17 . | 38 (14.3%) | 20 (18.0%) | 0.67 |
|  | Quadruple | 4 (1.1%) | 4 (1.3%) | 0 (0.0%) | 0.17 . | 4 (1.5%) | . | 0.67 |
|  | Debris comminuted | 8 (2.1%) | 6 (1.9%) | 2 (3.1%) | 0.17 . | 5 (1.9%) | 3 (3.0%) | 0.67 |
| **Postop complication** | No | 313 (83.0%) | 313 (100.0%) | – | – | 234 (88.3%) | 79 (71.0%) |  |
|  | Yes | 64 (17.0%) | – | 64 (100.0%) |  | 31 (11.7%) | 33 (29.0%) | <0.01 |
| **Wound healing disorder** | No | 352 (93.3%) | 313 (100.0%) | 39 (61.0%) |  | 256 (96.6%) | 96 (86.0%) | <0.01 |
|  | Yes | 25 (6.7%) | – | 25 (39.0%) |  | 9 (3.4%) | 16 (14.0%) |  |
| **Infection** | No | 344 (91.1%) | 313 (100.0%) | 31 (48.4%) |  | 249 (94.0%) | 95 (85.0%) | <0.01 |
|  | Yes | 33 (8.9%) | – | 33 (51.6%) |  | 16 (6.0%) | 17 (15.0%) |  |
| **Fistula** | No | 361 (95.8%) | 334 (100.0%) | 48 (75.0%) |  | 261 (98.5%) | 100 (89.0%) | <0.01 |
|  | Yes | 16 (4.2%) | – | 16 (25.0%) |  | 4 (1.5%) | 12 (11.0%) |  |
| **Plate exposure** | No | 362 (96.0%) | 313 (100.0%) | 49 (76.6%) |  | 259 (97.7%) | 103 (92.0%) | 0.02 |
|  | Yes | 15 (4.0%) | – | 15 (23.4%) |  | 6 (2.3%) | 9 (8.0%) |  |
| **Anticoagulation** | No | 349 (93.1%) | 295 (94.9%) | 54 (84.4%) |  | 241 (91.6%) | 108 (96.0%) | 0.07 |
|  | Yes | 26 (6.9%) | 16 (5.1%) | 10 (15.6%) | <0.01 | 22 (8.4%) | 4 (4.0%) |  |
| **Diabetes** | No | 359 (95.5%) | 298 (95.5%) | 61 (95.3%) |  | 251 (95.1%) | 108 (96.0%) |  |
|  | Yes | 17 (4.5%) | 14 (4.5%) | 3 (4.7%) | 1.00 | 13 (4.9%) | 4 (4.0%) | 0.61 |
| **Osteoporosis** | No | 366 (97.4%) | 306 (98.1%) | 60 (93.8%) |  | 255 (96.6%) | 111 (99.0%) | 0.29 |
|  | Yes | 10 (2.6%) | 6 (1.9%) | 4 (6.2%) | 0.06 | 9 (3.4%) | 1 (1.0%) |  |
| **Smoking** | No | 194 (58.6%) | 162 (60.0%) | 32 (53.3%) |  | 134 (58.5%) | 60 (59.0%) | 1.00 |
|  | Yes | 137 (41.4%) | 109 (40.0%) | 28 (46.7%) | 0.41 | 95 (41.5%) | 42 (41.0%) |  |
| **Alcohol** | No | 368 (97.6%) | 305 (97.4%) | 61 (98.4%) |  | 257 (97.0%) | 111 (99.0%) |  |
|  | Yes | 9 (2.4%) | 8 (2.6%) | 1 (1.6%) | 0.99 | 8 (3.0%) | 1 (1.0%) |  |

**Description**. Values are presented as absolute numbers with percentages or as mean ± standard deviation, as appropriate. Comparisons were performed between patients with and without postoperative surgical site– and hardware-related complications and between patients with and without osteosynthesis removal. Categorical variables were analyzed using the Chi-square test or Fisher’s exact test, and continuous variables using Student’s t-test or analysis of variance, as appropriate. Postoperative complications were defined as wound healing disorder, surgical site infection, fistula formation, or plate exposure. Sensory disturbances and postoperative pain were not included in the primary complication outcome. Missing data were handled by pairwise exclusion. Statistically significant p-values (p < 0.05) are highlighted.*, ^1^BMI = Body Mass Index (kg/m^2^), ^2^SD = Standard Deviation.*
